# Affectivity and satisfaction in the relationship of Pakistani couples is mediated by dyadic coping‐based gratitude

**DOI:** 10.1002/pchj.722

**Published:** 2023-12-27

**Authors:** Sultan Shujja, Adnan Adil

**Affiliations:** ^1^ Department of Psychology University of Sargodha Sargodha Pakistan; ^2^ Department of Psychology Government College Women University Sialkot Sialkot Pakistan

**Keywords:** context‐dependent gratitude, dyadic relationship satisfaction, trait affectivity

## Abstract

A substantial body of research supports a positive association between interpersonal gratitude and relationship satisfaction in couples; however, dyadic coping‐based gratitude (DC‐G) has not been investigated from a dyadic stress and coping perspective. The current study aimed to investigate the mediating role of DC‐G between trait affectivity and relationship satisfaction in couples. We collected data from both members of dyads (*N* = 300 married couples) for the study variables as a pre‐requisite for conducting dyadic data analysis using an actor–partner interdependent mediation model (APIMeM). The findings suggest that husbands' positive affect significantly predicted wives' relationship satisfaction via DC‐G (actor–partner effect). However, the mediating effect of DC‐G appeared to be stronger for the actor–actor and partner–partner effects compared with the cross‐partner effect, which supports the actor‐only effect. Further, wives' DC‐G mediated between husbands' negative affect and wives' relationship satisfaction, suggesting a mediating effect of DC‐G for wives but not for husbands. The implications are discussed within the context of couples' relationships.

## INTRODUCTION

Affectivity is a stable and enduring pattern of behaviors showing positive affect (PA), characterized by active, alert, attentive, determined, enthusiastic, excited, inspired, interested, proud, and strong attributes, and negative affect (NA), characterized by afraid, ashamed, distressed, guilty, hostile, irritable, jittery, nervous, scared, upset attributes, as opposed to the dimensions of a mood state (Watson et al., 2000; Watson & Tellegen, 1985; Zevon & Tellegen, 1982). Researchers who study relationships have reported a strong positive association between PA and relationship satisfaction, suggesting that positive traits provide a framework for the development and maintenance of stable and lasting relationships between couples (Ostovar et al., 2020). To date, relationship satisfaction has been studied specifically in terms of dyadic stress and coping (Badr et al., 2007; Falconier et al., 2015; Iqbal & Safdar, 2014; Lewandowski et al., 2010; Parise et al., 2017); that is, through marital satisfaction (Ostovar et al., 2020; Parise et al., 2019), romantic relationship satisfaction (Vollmann et al., 2019), and marital relationships (Salazar, 2015). However, the association between affectivity and relationship satisfaction has rarely been investigated with dyadic stress and coping as outcome variables (see Parise et al., 2019), for example, in the Systemic Transactional Model (STM; Bodenmann, 1997).

### An overview of the Systemic Transactional Model

The STM views stress as a systemic process, whereby the stress of one partner originating outside the romantic relationship (job‐related stress), and/or in the relationship (infidelity) crosses over to the other partner, leaving them stressed too. The process of stress and coping begins with the communication of stressful experiences with a non‐stressed partner. The non‐stressed partner engages in cognitive appraisal, evaluating the nature and severity of the stressful experiences. Following this, the non‐stressed partner can: discredit the partners' stress and hold them responsible for the stressful situation, disgracing them and helping them without motivation (negative dyadic coping [DC]); provide practical help (problem‐focused supportive DC); ensure emotional presence during the time of stress (emotion‐focused supportive DC); or take their partners' responsibilities on their shoulders (delegated DC). Additionally, both partners may make a joint effort to search for solutions in times of stress (common DC) (Bodenmann, 1997).

### Affectivity and relationship satisfaction within the stress and coping perspective

Researchers have demonstrated a significant positive association between PA and relationship satisfaction. Positive affectivity positively predicted relationship functioning (Gordon & Baucom, 2009; Watson et al., 2000). Owing to the strong positive association between positive affectivity and relationship satisfaction, PA was used as a control variable in a study that investigated the relationship between self‐concept clarity and relationship satisfaction in married couples, and the findings demonstrated that positive affectivity adds to the benefits of self‐concept clarity and increased relationship satisfaction for the dyads (Parise et al., 2019). Despite the positive association between affectivity and relationship satisfaction, little is known about the variables that provide a mechanism for explaining the improvement in the above‐mentioned relationship within the stress and coping paradigm, such as dyadic coping‐based gratitude (DC‐G).

### Affectivity, DC‐G, and relationship satisfaction

A recent set of studies defined DC‐G as a reaction of appreciation and thankfulness in response to problem‐focused and/or emotion‐focused positive DC behaviors by the partner (Shujja et al., 2022a), and this gratitude mediated between DC and relationship satisfaction in Pakistani couples (Shujja et al., 2022b).

To date, gratitude has been studied as a positive emotion associated with relationship satisfaction, relationship maintenance, and relationship commitment (Algoe, 2012; Algoe et al., 2016; Algoe & Zhaoyang, 2016; Leong et al., 2020; Vollmann et al., 2019). Studies have highlighted that the expression of gratitude is associated with increased PA, which in turn enhances couples' relationship satisfaction (Barton et al., 2015; Emmons et al., 2003). However, DC‐G, as a newly introduced construct (Shujja et al., 2022a), has not been studied in conjunction with affectivity and relationship satisfaction. Previous gratitude‐based studies (Barton et al., 2015; Emmons et al., 2003) treated positive affectivity as a general mood state, except for a single stress and coping study that included the trait affectivity as a control variable (Parise et al., 2019). The present study included trait affectivity as a predictor and tested its influence on the relationship satisfaction of couples via DC‐G.

Although gratitude towards a partner has been associated with relationship satisfaction (Algoe, 2012; Algoe & Zhaoyang, 2016; Vollmann et al., 2019), affectivity (positive or negative) has rarely been studied within the dyadic stress and coping literature (see Parise et al., 2019). Based on the existing literature, gratitude may act as a buffer against the initiation of negative feelings about the partner or the relationship and help to maintain relationship satisfaction (see Cassidy et al., 2009; Feeney & Van Vleet, 2010). Shujja et al. (2022b) argued that positive DC behaviors trigger DC‐G, which in turn mediates between couples' overall DC behaviors and relationship satisfaction. In general, gratitude has emerged as a mediator within relationship research, for example positive affectivity and relationship satisfaction (Unanue et al., 2019; Watkins et al., 2003). To our knowledge, only one study has investigated the mediating role of gratitude towards a partner between attachment styles and relationship satisfaction, and the findings revealed that attachment avoidance was associated with a lower level of gratitude towards a partner, which in turn predicted lower relationship satisfaction (Vollmann et al., 2019). However, DC‐G as a unique form of gratitude has not been tested as a variable that tends to explain the variance between trait affectivity and relationship satisfaction in Pakistani couples.

Based on the existing literature, this study aims to test the mediating role of DC‐G between trait affectivity and relationship satisfaction in Pakistani married couples. It is hypothesized that DC‐G will mediate the positive association between husbands' PA and their relationship satisfaction (actor effect) and that between husbands' PA and wives' relationship satisfaction (partner effect). Similarly, DC‐G is likely to act as a mediator between a negative association between husbands' NA and their relationship satisfaction (actor effect) and between husbands' NA and wives' relationship satisfaction (partner effect). To test the actor and partner mediating effects simultaneously, the Actor Partner Interdependence Mediation Model (APIMeM; Ledermann et al., 2011) was used.

## METHOD

### Participants and procedure

Pakistani couples who met the criteria, that is, married couples living together in Pakistan with a marriage duration of at least 1 year, were approached by posting invitations (Google Form link) on social media sites, namely Facebook, Twitter, WhatsApp, and emails, because physical access was minimal owing to the COVID‐19 pandemic. Before the study, the potential participants were asked to give written informed consent by clicking the “I Agree to” tab after carefully reading the information regarding potential benefits, risks, purpose, study procedure, rights and responsibilities of the participants and researcher, privacy, and confidentiality of the data. We asked the participants to provide partners' contact details, such as email, after getting the partners' permission for sharing contact information, and the exchange of partners' information was set as a pre‐requisite for the inclusion of participants' responses in the final data set and for obtaining the couples' data. Along with a demographic sheet, respondents were asked to fill in the study questionnaires online, and data were automatically recorded in an Excel sheet, which was later imported to the IBM SPSS statistics for windows (version 25) data file. The Ethical Research Committee of the Department of Psychology at the University of Sargodha, Pakistan approved the study to ensure that it was carried out in strict adherence with the ethical guidelines set by the American Psychological Association (APA, 2022). All participants gave their written informed consent to participate in the present study on a voluntary basis. As a result, 955 participants agreed to participate and responded to the study questionnaires along with the demographic sheet. Following the data screening, 355 respondents were eliminated from the final dataset, including 179 for not providing partners' contact information, 123 for leaving the Google Form incomplete, and 53 for not meeting the inclusion criterion (1‐year marriage duration).

The final sample comprised 300 Pakistani couples (*N*_Husbands_ = 300; *N*_wives_ = 300), with the husbands' ages between 20 and 79 years (*M* = 41.59 years, *SD* = 10.96) and the wives' ages between 20 and 65 years (*M* = 37.16 years, *SD* = 9.55). In the current sample, we labeled the husband as an actor and the wife as a partner within a dyad. Marriage duration had a mean of 10.44 years (SD = 8.66 years). Out of the total sample, 51.2% of couples lived in urban areas of the country, while 48.8% lived in the countryside. Also, 56% of couples reported that they were living within a nuclear family system, while 43.5% lived in an extended family system. Further, 76.8% of couples reported that they got married through the arranged marriage system (parents decide the life partner for their children), while 23.2% had a love marriage.

### Measures

#### Positive and negative affect

The Positive and Negative Affect Schedule (PANAS; Watson et al., 1988) has been designed to measure affectivity as a trait. It contains 20 items, including 10 PA items and 10 NA items as clearly distinct subscales reported in the original version of PANAS. Respondents rate themselves on a 5‐point Likert‐type response format ranging from (1) *very slightly or not at all* to (5) *extremely*, and scores on particular subscales range from 10 to 50. Respondents scoring high on a particular subscale (PA or NA) are treated as high on that particular trait and vice versa. The Urdu translation of PANAS shows good psychometric properties (Niazi, 2017) and has been used in the current study with good internal consistency measure for the PA (*α* = .73) and the NA (*α* = .78).

##### Dyadic coping‐based gratitude questionnaire

Developed by Shujja et al. (2022a), the dyadic coping‐based gratitude questionnaire is a self‐report measure designed to assess expressed gratitude following partners' positive dyadic coping (DC) behaviors. It contains 12 items, namely 6 items for the partner (partners' expression of gratitude in response to the non‐stressed partners' DC in the time of stress) and 6 items for the self (respondents' expression of gratitude in response to the non‐stressed partners' DC in the time of stress). Out of the 6 items, three pertain to problem‐focused DC‐based gratitude and three to emotion‐focused DC‐based gratitude for the partner and the same two factors for the self. Sample items for the partner are *My partner shows gratitude towards me, by telling me, writing me a note, or giving me a gift when I have instrumentally supported him/her when he/she has been stressed* (problem‐focused dyadic coping‐based gratitude); *My partner expresses gratitude for my emotional support* (e.g., *listening, empathy, understanding, and helping to regulate his/her emotions*) *during times when he/she has felt stressed* (emotion‐focused dyadic coping‐based gratitude), and for the self are *I tell my partner that I value his/her practical guidance and advice when I have been stressed* (problem‐focused dyadic coping‐based gratitude); *I express gratitude towards my partner for his/her emotional support* (e.g., *listening, empathy, understanding, helping to regulate his/her emotions*) *during times when I have felt stressed* (emotion‐focused dyadic coping‐based gratitude). Participants responded in a 5‐point Likert‐type response format ranging from *never* (1) to *very often* (5), and the mean scores on the scale were used as an index of DC‐based gratitude. Respondents scoring high on the DC‐GQ were treated as high on expressed gratitude following partners' DC behaviors and vice versa. Psychometric properties show that DC‐GQ is a valid and reliable self‐report measure for the Pakistani married adults, and internal consistency measures for the current study are reported in Table 1.

**TABLE 1:** Descriptive statistics along with the paired sample *t* test and correlation matrix for the study variables (*N* = 300 couples).

Variables	Husbands (A)	Wives (P)	Paired *t*	α Husbands	α Wives	Correlations			
	*M* (*SD*)	*M* (*SD*)				1	2	3	4
1 PA	3.12 (0.64)	3.04 (0.61)	1.91*	.74	.71	**.33** ***	−.15**	.20**	.12*
2 NA	2.36 (0.61)	2.43 (0.62)	2.10*	.79	.77	−.20**	**.42** ***	−0.06	−.28***
3 DC‐G	3.34 (0.87)	3.29 (0.86)	1.06	.91	.90	.20**	−0.05	**.55** ***	.34***
4 RS	3.67 (0.70)	3.70 (0.72)	.64	.77	.78	.24***	−.19**	.37***	**.53** ***

*Note*: Correlation coefficients above the diagonal pertain to husbands (A = actor) and those below the diagonal pertain to wives (P = partner). Correlation coefficients reported in boldface represent husbands’ and wives' correlation on the study variables.

Abbreviations: DC‐G = dyadic coping‐based gratitude; NA = negative affect; PA = positive affect; RS = relationship satisfaction.

* *p* < .05;

** *p* < .01;

*** *p* < .001.

##### Relationship assessment scale

Developed by Hendrick (1988), the relationship assessment scale (RAS) is a one‐dimensional self‐report measure designed to assess the level of relationship satisfaction in a relationship with a partner. It contains 7 items with a 5‐point Likert‐type response format ranging from 1 (*low satisfaction*) to 5 (*high satisfaction*). Sample items are *In general, how satisfied are you with your relationship?* and *How good is your relationship compared to most?* For the current study, the Urdu translation of the RAS (Shahid, 2014) shows good psychometric properties, and internal consistency measures (alpha reliability estimates) are reported in Table 1.

## RESULTS

### Data analyses

Initially, dyadic data were screened to identify the missing values, error, omissions, and other irregularities during the data entry, such as faulty mean values etc. Descriptive statistics (mean, standard deviation) were computed along with a paired sample *t* test to gain an overall picture of the dyadic dataset. Further, correlation analyses were conducted to assess the level of interdependence in the responses of dyads, and interdependence led towards the violation of the condition of independent observation for parametric tests such as hierarchical regression. Kenny et al. (2006) suggested a test of distinguishability for the dyadic dataset to test the null hypothesis that sex would not act as a distinguishing variable on the outcome variable (relationship satisfaction). In order to test the null hypothesis, −2 log‐likelihood estimation was used to assess the difference in variance between compound symmetry (CS) and heterogeneous compound symmetry (CSH) variance types. The chi‐square difference test (*χ* ^2^ [1] = 0.22, *p* > .05) demonstrates that sex did not emerge as a distinguishing variable for the outcome variable, and the dyad members were found to be indistinguishable for the current study. To account for the indistinguishability, the dataset was restructured into a person period pairwise dataset (Kenny et al., 2006), in which data for each study variable are entered twice, once for the actor (A) and once for the partner (P). To test the mediating role of DC‐G in the association between PA/NA and relationship satisfaction, one set of data (husbands' dataset) was subjected to the Actor Partner Interdependence Mediation Model (APIMeM), which has been reported as the most appropriate statistical analysis for a dyadic dataset (Ledermann et al., 2011). By employing the maximum likelihood bootstrap method, we generated 5000 bootstrapped samples to calculate bias‐corrected 95% confidence intervals for the indirect effects.

#### Descriptive statistics

Means, standard deviations, and correlations are presented in Table 1. Husbands' high score on PA demonstrated that husbands were generally engaged in showing more PA compared with their wives, whereas wives showed more NA compared with their husbands. Further, no sex differences have been reported on DC‐G, and relationship satisfaction and reliability estimates on the study variables for the husbands and wives remain within the acceptable range (0.71–0.91). The correlation matrix shows the values for the husbands above the diagonal, and those for the wives below the diagonal, and boldface values indicate husbands' and wives' correlation on the study variables. As expected, NA is negatively and significantly correlated with all the study variables except DC‐G for the husbands and wives. These results suggest that husbands and wives with high NA are less likely to experience PA and relationship satisfaction. In contrast, PA is associated with a high level of DC‐G and relationship satisfaction. The boldface correlation coefficients reveal non‐independence (interdependence) between husbands and wives (*r* = 0.33–0.55) in the study, justifying dyadic analyses of the data.

**FIGURE 1:**
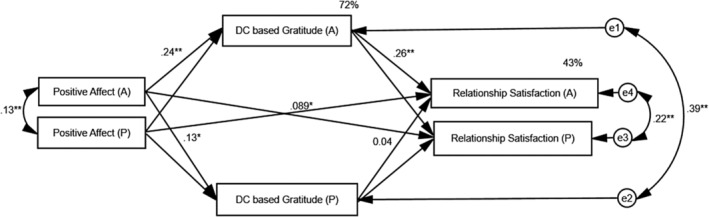
Path diagram showing the mediating role of dyadic coping‐based gratitude (DC‐G) between positive affect (PA) and relationship satisfaction in couples. Dyadic coping is treated as a control variable. Only one set of data was used because the dyad was treated as indistinguishable. *p < .01, **p < .001.

**FIGURE 2:**
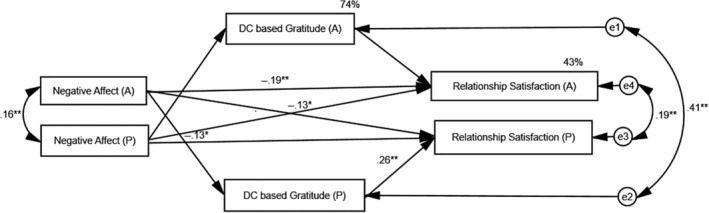
Path diagram showing the mediating role of dyadic coping‐based gratitude (DC‐G) between negative affect (NA) and relationship satisfaction in couples. Dyadic coping is treated as a control variable. Only one set of data was used because the dyad was treated as indistinguishable. *p < .01, **p < .001.

The APIMeM (Ledermann et al., 2011) was used to compare the model with PA as a predictor with the model using NA as a predictor, and the chi‐square difference test, *χ* ^2^ (1, 300) = 20, *p* < .01 demonstrated a significant difference between the two models. Given this, two separate mediation analyses were conducted for the measurement models. APIMeM served three objectives: (a) accounting for the variability due to interdependence between the dyad members; (b) measuring one partners' predictor and mediator on the dyadic outcomes; and (c) measuring residual covariance between variable pairs. In the current study, one set of coefficients is presented owing to the evidence for the indistinguishability between the dyad members and the restructured person period pairwise data set (see Figures 1 and 2).

### Testing the direct effects of PA on relationship satisfaction

A saturated model (*df* = 0) with PA as predictor demonstrates the direct actor effect (PA_A_→relationship satisfaction_A_) to be nonsignificant. This shows the complete mediation for the direct actor effect (for a review, see Kenny et al., 2006). To test for complete mediation, we removed the above‐mentioned direct path from the measurement model and re‐ran the analysis following model re‐specification. The model fit indices indicate a good fit (Chi square [*χ* ^2^ ] [2] = 6.8, *p >* .05; Comparative fit index [CFI] = 0.99; Goodness of fit index [GFI] = 0.99; Root mean square error of approximation [RMSEA] = 0.06; Standardized Root Mean Squared Residual [SRMR] = 0.02). All the direct actor and partner effects remain significant except the partner effect of DC‐G on relationship satisfaction. Further, the direct actor effect is positive and greater in size than the corresponding partner effect (see Figure 1), suggesting that husbands' higher level of PA is associated with a higher level of DC‐G than that of wives' PA, and that husbands' higher level of DC‐G is associated with the husband and partners' relationship satisfaction. These results support the actor‐only pattern (Kenny & Cook, 1999). PA accounts for the 72% variance in couples' DC‐G, which, in turn, accounts for 44% of the variance in explaining the relationship satisfaction.

### Testing the indirect effect with PA on relationship satisfaction

For the APIMeM with indistinguishable dyads, there are three specific indirect paths, namely actor–actor effect, partner–partner effect, and actor–partner effect. All the paths have been tested for significance to determine the mediating role of DC‐G, and the estimates are listed in Table 2. The estimates of specific indirect paths support our hypothesis suggesting that DC‐G mediates the association between PA and husbands' own and wives' relationship satisfaction. Although the actor–partner specific indirect effect showed significant mediation, the actor–actor effect or partner–partner effect more strongly contributes to the complete mediation in the corresponding direct paths (e.g., PA_A_→relationship satisfaction_A_), indicating that husbands' high PA is associated with a high level of husbands' own and wives' relationship satisfaction when DC‐G mediates the relationship.

**TABLE 2:** Specific indirect effects considering couples as indistinguishable with positive and negative affect as predictors, DC‐based gratitude as mediator, and relationship satisfaction as the outcome variable (*N* = 300).

Paths	Β	*p*	95% CI
PA_A_ → DC‐G_A_ → Relationship satisfaction_A_	0.062	.001	0.030–0.104
PA_A_ → DC‐G_A_ → Relationship satisfaction_P_	0.010	.28	−0.009–0.034
PA_A_ → DC‐G_P_ → Relationship satisfaction_A_	0.005	.19	−0.003–0.023
PA_A_ → DC‐G_P_ → Relationship satisfaction_P_	0.034	.02	0.004–0.069
NA_A_ → DC‐G_P_ → Relationship satisfaction_P_	−0.034	.01	−0.062– −0.010

*Note*: Only actor (husband) estimates for specific indirect effects are reported owing to the indistinguishable status of the dyads.

Abbreviations: A = actor; P = partner.

### Testing the direct effects of NA on relationship satisfaction

The saturated model with just identified parameters (*df* = 0) demonstrates all the direct paths to be significant except the actor effect (NA_A_→DC‐G_A_) with and partner effect (DC‐G_A_→relationship satisfaction_P_). After the removal of the nonsignificant direct paths, we re‐ specified the measurement model, and model fit indices show a good model fit (*χ* ^2^ [2] = 3.39, *p* = .49; CFI = 1.000; GFI = 0.99; RMSEA = 0.000; SRMR = 0.018). The re‐specified model shows that NA_A_ negatively predicts DC‐G_P_, suggesting that a higher level of NA_A_ is associated with low DC‐G_p_; however, DC‐G_A_ positively and significantly predicts ones' own relationship satisfaction, not that of partners. The NA_A_ has emerged as a stronger negative predictor of ones' own relationship satisfaction (*β* = −.17, *p* < .001; 95% CI = −.094– −.25) compared with the NA_P_ (*β* = −.11, *p* < .01, 95% CI= −.03– −.197), indicating a stronger association between NA and relationship satisfaction for the actor effect than for the partner effect (see Figure 2).

### Testing the specific indirect effects of NA on relationship satisfaction

In the model with NA as a predictor, the specific indirect paths, that is, NA_A_→DC‐G_A_→relationship satisfaction_A_ (one path nonsignificant) and NA_A_→DC‐G_A_→relationship satisfaction_P_ (both paths nonsignificant), have not been tested for significance because one or both nonsignificant paths imply the absence of mediation (for a review, see Ledermann & Macho, 2009). The only indirect path, that is, NA_A_→DC‐G_P_→relationship satisfaction_P_, has been tested for significance, and the results are listed in Table 2. The results support our hypothesis that DC‐G would mediate the negative association between NA and relationship satisfaction in dyad members. This means that higher NA predicts low relationship satisfaction, and that the inclusion of DC‐G as a mediator in the measurement model (see Figure 2) explains the negative association between husbands' NA and wives' relationship satisfaction.

## DISCUSSION

The current study has examined the way that DC‐G operates between trait affectivity and relationship satisfaction in Pakistani couples. Previously, positive affectivity has been reported to positively predict relationship satisfaction in couples (Parise et al., 2019; Watson et al., 2000), and the findings of the current study are in line with that literature. This suggests that couples with higher PA tend to cope better with dyadic stress and strive for enhanced relationship satisfaction. Additionally, PA may be associated with a high level of interdependence between the couple, having them feel more positively about each other and the relationship, ultimately leading to increased relationship satisfaction. Given this, Fosha (2001) emphasized the dyadic nature of affectivity and couples' capacity to regulate each other's positive mood state. Moreover, another construct as demonstrated in the current study, namely DC‐G (Shujja et al., 2022b), significantly mediates the association between PA and relationship satisfaction in Pakistani couples, suggesting couples' high relationship satisfaction following the inclusion of DC‐G in the measurement model compared with the direct association between PA and relationship satisfaction without the inclusion of DC‐G as a mediator (see Figure 1). The mediating role of DC‐G in the measurement model provides evidence for the presence of DC‐G as a significant variable for a high level of couples' relationship satisfaction in addition to PA.

Previously, interpersonal gratitude has been studied as generally useful in helping romantic relationships to flourish (Algoe, 2012; Gottman, 1994), whereas DC‐G is a context‐dependent form of gratitude that originates in particular after a partner's positive DC behaviors, and it needs attention from a dyadic stress and coping perspective. It seems reasonable to attribute the strengthened association between PA and relationship satisfaction to DC‐G because gratitude in general raises the recipient's positive mood state and positive opinion about their partner and the relationship (Algoe, 2012), and DC‐G enhances couples' PA and relationship satisfaction in particular.

In contrast, negative affectivity is negatively associated with relationship satisfaction, and our findings are in line with the existing literature (Gana & Jakubowska, 2016; Rehman et al., 2008; Renshaw et al., 2010). The findings of the current study demonstrate that husbands' (actor) NA significantly crosses over to the wives' relationship satisfaction, reflecting the dyadic influence of NA, and these findings are in line with the existing literature (see Renshaw et al., 2010). These findings provide partial support for our hypothesis about the mediating role of DC‐G between husbands' NA and relationship satisfaction (actor effect) and husbands' NA and wives' relationship satisfaction (partner effect). The current study provided support for the partner effect but not for the actor effect, suggesting that the impacts of NA strongly cross over to the partner's relationship satisfaction. This means that DC‐G acts as a mediator between the negative association NA_A_ and relationship satisfaction_P_, and the association tends to improve as the DC‐G mediates the measurement model. The significance of the DC‐G as a mediator stands firm with the interpersonal relationship, as it intervenes positively in the association between PA/NA and relationship satisfaction in Pakistani couples.

This study has wide implications for marriage and family counselors, as they can use DC‐G as a source for boosting couples' PA and relationship satisfaction. Further, interventions such as brief gratitude‐based interventions (see Kanter & Schramm, 2018) may be devised to improve the negative association between couples' NA and relationship satisfaction and to strengthen the relationship between couples' PA and relationship satisfaction. Additionally, interpersonal researchers in Pakistan may confidently consider trait affectivity as a control variable, knowing that it can confound interpersonal variables and consequent outcomes, as was done by Parise et al. (2019) in investigating the association between self‐concept clarity and relationship satisfaction. This study undoubtedly contributes to an understanding of couples' trait affectivity and relationship satisfaction using dyadic data with a special focus on a new form of gratitude (i.e., DC‐G; Shujja et al., 2022a).

However, this study is not without limitations. First, cross‐sectional data do not provide evidence for the temporal validity of the mediation findings. For this, we suggest extending this study using longitudinal data to understand the meditational impact over time. Second, the data set includes heterosexual Pakistani married couples but speaks less about other types of couples, such as same‐sex couples, dating couples, and cohabitating couples, and further research should include an ethnically, demographically, and culturally diverse sample for expansion of scientific knowledge on the role of DC‐G. Third, the quantitative dataset restricts us to making an ideographic analysis, which requires qualitaitve and open‐ended responses to understand lived expereinces of the participants. Future research may extend this effort by including a qualitative dataset for an extensive exploration of the phenomenon.

In short, the association between trait affectivity and relationship satisfaction has been investigated by introducing DC‐G as a mediator within the measurement model for Pakistani married couples. By treating the dyad (husband and wife) as indistinguishable and using the APIMeM (Ledermann et al., 2011), we tested the mediation models for the actor (husband) effect and the partner (wife) effect. The findings demonstrate that PA significantly predicts couples' relationship satisfaction via DC‐G for the actor–actor or partner–partner effect and the actor–partner effect, suggesting that husbands’ and wives' PA significantly predicts their own (actor–actor/partner–partner) relationship satisfaction and partners' relationship satisfaction (actor–partner) via DC‐G; however, mediation turns out to be stronger for the actor–actor/partner–partner effect than for the actor–partner effect. For negative affectivity, the actors' NA on the partners' relationship satisfaction via the mediating role of DC‐G has emerged as the only significant path in the measurement model, suggesting a significant actor–partner indirect effect. This suggests that the wives' DC‐G acts as a mediator in the negative association between husbands' NA and wives' relationship satisfaction and that a negative relationship improves owing to the mediating role of the DC‐G.

## CONFLICT OF INTEREST STATEMENT

The authors declare no conflicts of interest.

## ETHICS STATEMENT

The research was carried out in strict compliance with the ethical guidelines of the American Psychological Association and was monitored by the Research Ethics Committee of the Department of Psychology, University of Sargodha.

## Data Availability

The data that support the findings of this study are available from the corresponding author upon reasonable request.
